# Testosterona, Níveis de SHBG e Risco de Insuficiência Cardíaca: a Correlação Implica Causalidade?

**DOI:** 10.36660/abc.20250593

**Published:** 2025-11-27

**Authors:** Érique José Farias Peixoto de Miranda

**Affiliations:** 1 Centro de Ensaios Clínicos e Farmacovigilância, Instituto Butantan São Paulo SP Brasil Centro de Ensaios Clínicos e Farmacovigilância, Instituto Butantan (IB), São Paulo, SP – Brasil

**Keywords:** Insuficiência cardíaca, Terapia de Reposição Hormonal, Testosterona

Os autores descrevem uma metanálise^1^ de seis estudos observacionais com baixo risco de viés, que sugere uma associação modesta entre baixos níveis de testosterona, mas não de Globulina Ligadora de Hormônios Sexuais (SHBG), e a incidência de insuficiência cardíaca (IC) em homens, mas não em mulheres. Em resumo, para cada redução de um desvio padrão nos níveis séricos de testosterona, a razão de risco de IC aumentou 10% (HR 1,10, IC 95%: 1,03-1,17).

Os dados coletados mostram que um total de 233.474 participantes de meia-idade foram acompanhados por pelo menos 7,7 anos; a grande maioria deles (90,2%) se registrou em uma única coorte,^2^ 3.991 (1,71%) deles apresentaram eventos. Em outras palavras, observamos um pequeno aumento no risco absoluto em comparação com a população em geral.^3^ Entre indivíduos com idade ≥ 45 anos, a incidência de IC é de 6,0 por 1.000 pessoas-ano, aumentando para 21,1 por 1.000 pessoas-ano entre aqueles com 65 anos ou mais.^4^

Portanto, devemos encorajar a reposição de testosterona para qualquer homem com mais de 40 anos ou apenas para aqueles com hipogonadismo hipogonadal para prevenir a IC? A metanálise avaliou a associação, mas não a causalidade. Apesar das inconsistências, estudos epidemiológicos identificaram vias entre hormônios sexuais (SHs) e IC, já que o declínio relacionado à idade nesses hormônios resulta em mudanças fisiológicas que impactam o risco de IC não apenas em homens, mas também em mulheres,^5^ apesar dos resultados da metanálise. Apesar da associação entre baixos níveis de testosterona e IC em homens, baixos níveis de SHs em homens de meia-idade podem ser um marcador de saúde geral precária (por exemplo, má nutrição, estilo de vida sedentário, obesidade ou comorbidades, como hipertensão, dislipidemia e diabetes), mais fortemente correlacionados com o risco cardiovascular em si, em vez de uma causa direta da IC.^5^ Além disso, os ciclos de feedback dos SHs são interrompidos na IC.^5^

No entanto, os próprios autores destacam que as evidências são fracas (fator de Bayes de 0,99), indicando que a chance de a hipótese nula (ausência de associação) ser verdadeira é quase a mesma que a hipótese alternativa. A relevância clínica desse achado é discutível e certamente não justifica uma recomendação de suplementação de testosterona em larga escala.

Além disso, a metanálise teve uma seleção muito rigorosa, possivelmente devido à falta de estudos de alta qualidade que mediram a testosterona basal e monitoraram os resultados da IC. Apesar disso, os autores consideram que a chance de viés de publicação não é significativa. Além disso, a heterogeneidade dos estudos (devido a diferentes populações, métodos de medição hormonal, etc.) é outro fator limitante, conforme apontado pelos próprios autores, e pode influenciar a precisão dos resultados. Ebong et al. reconhecem que uma abordagem padronizada para a medição da SH é necessária para uma comparação confiável entre os estudos.^5^

Os achados referentes às mulheres neste estudo refletem a controvérsia na literatura.^6^ Embora a menopausa e a redução dos níveis de estrogênio estejam associados a um risco cardiovascular aumentado em mulheres, os estudos sobre terapia de reposição hormonal (TRH) em mulheres são complexos e controversos. O estudo da *Women's Health Initiative* de 2002,^6^ por exemplo, demonstrou que a TRH combinada (estrogênio e progesterona) aumentou o risco de eventos cardiovasculares, acidente vascular cerebral, tromboembolismo venoso e câncer de mama, o que levou a um declínio drástico nas prescrições de TRH. Portanto, a motivação para a TRH em mulheres é principalmente aliviar os sintomas da menopausa e não prevenir a IC.

Os autores reconhecem a necessidade de ensaios clínicos randomizados para explorar a relação entre os níveis de testosterona e a IC. Poucos ensaios clínicos randomizados, duplo-cegos e controlados por placebo que avaliaram a terapia de reposição de testosterona (TRT) em homens de meia-idade não incluíram a eficácia da prevenção de eventos cardiovasculares adversos maiores (MACE) como desfecho primário, mas sim a segurança da reposição hormonal.^7,8^ Os MACE foram um composto de morte cardiovascular, infarto do miocárdio não fatal e acidente vascular cerebral não fatal.

A TRT para Eventos Vasculares em Avaliação Estruturada (TRAVERSE)^7^ foi um ensaio clínico de não-inferioridade que incluiu quase 5.000 homens com idades entre 45 e 80 anos com hipogonadismo e histórico ou alto risco de doença cardiovascular. Este estudo avalia a segurança cardiovascular da TRT, especificamente sua não inferioridade ao placebo em relação à incidência de MACE. O acompanhamento médio foi de 33,0 ± 12,1 meses. O estudo concluiu que a TRT não foi inferior ao placebo em relação à incidência de MACE, ou em outras palavras, a TRT não aumentou o risco cardiovascular nessa população de alto risco. Apesar dos resultados tranquilizadores obtidos no TRAVERSE, Basaria et al. ^8^ mostraram que entre 209 homens (idade média de 74 anos) com mobilidade limitada, hipogonadismo e doença cardiovascular preexistente ou de alto risco. No entanto, este estudo foi interrompido precocemente devido a preocupações com a segurança, sugerindo um risco aumentado de eventos cardiovasculares adversos, como infarto do miocárdio.

Portanto, não devemos incentivar a suplementação de testosterona para homens com ou sem hipogonadismo para a prevenção da IC. No entanto, a modesta associação encontrada na metanálise enfatiza a necessidade de ensaios clínicos randomizados com potência para explorar a relação entre os níveis de testosterona e os MACE.

## References

[1] Artioli T, Batista LB, Franchini K, Alencar JN (2025). Impact of Low Testosterone and SHBG Levels on Heart Failure Risk: A Systematic Review and Meta-Analysis. Arq Bras Cardiol.

[2] Yeap BB, Marriott RJ, Antonio L, Raj S, Dwivedi G, Reid CM (2022). Associations of Serum Testosterone and Sex Hormone-Binding Globulin with Incident Cardiovascular Events in Middle-Aged to Older Men. Ann Intern Med.

[3] Khan MS, Shahid I, Bennis A, Rakisheva A, Metra M, Butler J (2024). Global Epidemiology of Heart Failure. Nat Rev Cardiol.

[4] Huffman MD, Berry JD, Ning H, Dyer AR, Garside DB, Cai X (2013). Lifetime Risk for Heart Failure among White and Black Americans: Cardiovascular Lifetime Risk Pooling Project. J Am Coll Cardiol.

[5] Ebong IA, Appiah D, Mauricio R, Narang N, Honigberg MC, Ilonze OJ (2025). Sex Hormones and Heart Failure Risk. JACC Adv.

[6] Rossouw JE, Anderson GL, Prentice RL, LaCroix AZ, Kooperberg C, Stefanick ML (2002). Risks and Benefits of Estrogen Plus Progestin in Healthy Postmenopausal Women: Principal Results from the Women's Health Initiative Randomized Controlled Trial. JAMA.

[7] Lincoff AM, Bhasin S, Flevaris P, Mitchell LM, Basaria S, Boden WE (2023). Cardiovascular Safety of Testosterone-Replacement Therapy. N Engl J Med.

[8] Basaria S, Coviello AD, Travison TG, Storer TW, Farwell WR, Jette AM (2010). Adverse Events Associated with Testosterone Administration. N Engl J Med.

