# Supplementary material for: Testosterona, Níveis de SHBG e Risco de Insuficiência Cardíaca: a Correlação Implica Causalidade?
Source: Arq Bras Cardiol. 2025 Nov 27;122(10):e20250593. [Article in Portuguese] doi: 10.36660/abc.20250593 (PMC12671722; doi:10.36660/abc.20250593)
Supplement: Translation

# Translation: Testosterone, SHBG Levels, and Heart Failure Risk: Does the Correlation Imply Causation?

**DOI:** 10.36660/abc.20250593i

**Authors:** Érique José Farias Peixoto de Miranda

**Affiliations:** 1 São Paulo SP Brazil Centro de Ensaios Clínicos e Farmacovigilância, Instituto Butantan (IB), São Paulo, SP – Brazil

**Keywords:** Heart Failure, Hormone Replacement Therapy, Testosterone

The authors describe a meta-analysis^1^ of six observational studies with low risk of bias, which suggests a modest association between low testosterone levels, but not Sex Hormone-Binding Globulin (SHBG), and incidence of heart failure (HF) in men, but not in women. In short, for each reduction of one standard deviation of serum testosterone levels, the hazard ratio of HF increased 10% (HR 1.10, 95% CI: 1.03-1.17).

The polled data show that a total of 233,474 middle-aged participants were followed up for at least 7.7 years; the vast majority of them (90.2%) registered in one single cohort,^2^ 3,991 (1.71%) of them had events. In other words, we could observe a small absolute risk increase in comparison to the overall population.^3^ Among individuals aged ≥45 years, the incidence of HF is 6.0 per 1,000 person-years, increasing to 21.1 per 1,000 person-years among those aged 65 years or more.^4^

Therefore, should we encourage testosterone replacement for any man over 40 or only for those with hypogonadal hypogonadism to prevent HF? The meta-analysis assessed association, but not causation. Despite the inconsistencies, epidemiological studies identified pathways between sex hormones (SHs) and HF, as the age-related decline in those hormones results in physiological changes that impact HF risk not only in men, but also in women,^5^ despite the results of the meta-analysis. Despite the association between low testosterone levels and HF in men, low levels of sexual hormones in middle-aged men may be a marker of poor general health (e.g., poor nutrition, sedentary lifestyle, obesity, or comorbidities, like hypertension, dyslipidemia, and diabetes), more strongly correlated with cardiovascular risk itself, rather than a direct cause of HF.^5^ Besides that, SHs feedback loops become disrupted in HF.^5^

Nevertheless, the authors themselves highlight that the evidence is weak (Bayes factor of 0.99), indicating that the chance of the null hypothesis (no association) being true is almost the same as the alternative hypothesis. The clinical relevance of this finding is debatable, and certainly does not justify a recommendation for large-scale testosterone supplementation.

Furthermore, the meta-analysis had a very strict selection, possibly due to the lack of high-quality studies that measured baseline testosterone and monitored HF outcomes. Despite that, the authors consider that the chance of publication bias is not significant. Besides that, the heterogeneity of the studies (due to different populations, hormone measurement methods, etc.) is another limiting factor, as pointed out by the authors themselves, and may influence the accuracy of the results. Ebong et al. recognize that a standardized approach to SH measurement is needed for reliable comparison across studies.^5^

The findings regarding women in this study mirror the controversy in the literature.^6^ Although the menopause and declining estrogen levels are associated with an increased cardiovascular risk in women, the studies on hormone replacement therapy (HRT) in women are complex and controversial. The 2002 Women's Health Initiative study,^6^ for example, demonstrated that combined HRT (estrogen and progesterone) increased the risk of cardiovascular events, stroke, venous thromboembolism, and breast cancer, which led to a dramatic decline in HRT prescriptions. Therefore, the motivation for HRT in women is primarily to alleviate menopausal symptoms and not to prevent HF.

The authors acknowledge the need for randomized clinical trials to explore the relationship between levels of testosterone and HF. Few randomized, double-blind, placebo-controlled clinical trials that evaluated the Testosterone Replacement Therapy (TRT) in middle-aged men did not include efficacy of prevention of Major Adverse Cardiovascular Events (MACE) as the primary outcome, but safety of hormone replacement.^7,8^ MACE was a composite of cardiovascular death, non-fatal myocardial infarction, and non-fatal stroke.

The Testosterone Replacement Therapy for Vascular Events in Structured Evaluation (TRAVERSE)^7^ was a non-inferiority clinical trial that included nearly 5,000 men aged 45 to 80 with hypogonadism and a history or high risk of cardiovascular disease. This study evaluates the cardiovascular safety of TRT, specifically its non-inferiority to placebo regarding the incidence of MACE. Mean follow-up was 33.0±12.1 months. The study concluded that TRT was non-inferior to placebo regarding the incidence of MACE, or in other words, TRT did not increase cardiovascular risk in this high-risk population. Despite the reassuring results obtained in TRAVERSE, Basaria et al.^8^ showed that among 209 men (mean age, 74 years) with limited mobility, hypogonadism, and preexisting or a high risk of cardiovascular disease. However, this study was stopped early due to safety concerns, suggesting an increased risk of adverse cardiovascular events, such as myocardial infarction.

Thus, we should not encourage testosterone supplementation for any men with or without hypogonadism for the prevention of HF. However, the modest association found in the meta-analysis emphasizes the need for powered randomized clinical trials to explore the relationship between testosterone levels and MACE.
